# Supplementary material for: The morphometrical and topographical evaluation of the superior gluteal nerve in the prenatal period
Source: PLoS One. 2022 Aug 26;17(8):e0273397. doi: 10.1371/journal.pone.0273397 (PMC9417028; doi:10.1371/journal.pone.0273397)
Supplement: Attachment — Submitted filename: Response to Reviewers.doc [file pone.0273397.s001.doc]

Dear Reviewer,

We have incorporated all of your suggestions into our revision, except part of suggestion 4 concerning decimal place for mathematical accuracy ("The mean and median ought to be presented with one decimal place (SD with two) for mathematical accuracy.").

After discussing with our statistician, we ask you to accept our version of the decimal places for the mean and median, because the number of decimal places depends on the accuracy of the measurement of the physical quantity. When measuring the length of biological structures (in millimeters), the actual average should be recorded to one decimal place. A measure of variation, i.e. standard deviation (SD), is always given in the same units with the same accuracy, so one decimal place is also enough.

Thank you for your help.

Kind regards

Wojciech Derkowski, MD, PhD
