# Supplementary material for: The morphometrical and topographical evaluation of the superior gluteal nerve in the prenatal period
Source: PLoS One. 2022 Aug 26;17(8):e0273397. doi: 10.1371/journal.pone.0273397 (PMC9417028; doi:10.1371/journal.pone.0273397)
Supplement: Attachment — Submitted filename: Response to Reviewers.doc [file pone.0273397.s002.doc]

Dear Reviewer,

We have incorporated all of your suggestions into our revision.

We have added section "limitations of the study" as the last paragraph of the discussion.

Thank you for your help.

Kind regards

Wojciech Derkowski, MD, PhD
