# Supplementary material for: The morphometrical and topographical evaluation of the superior gluteal nerve in the prenatal period
Source: PLoS One. 2022 Aug 26;17(8):e0273397. doi: 10.1371/journal.pone.0273397 (PMC9417028; doi:10.1371/journal.pone.0273397)
Supplement: Attachment — Submitted filename: Response to Reviewers.doc [file pone.0273397.s003.doc]

Dear Reviewer,

We have incorporated all of your suggestions into our revision.

We have changed in table 2 Fisher exact test for chi2 with Yates correction.

Thank you for your help.

Kind regards

Wojciech Derkowski, MD, PhD
